# Characterization of a rabbit osteoporosis model induced by ovariectomy and glucocorticoid

**DOI:** 10.3109/17453674.2010.483986

**Published:** 2010-05-21

**Authors:** Li Baofeng, Yuan Zhi, Chen Bei, Meng Guolin, Yin Qingshui, Liu Jian

**Affiliations:** ^1^Department of Orthopaedics, Guangzhou General Hospital of Guangzhou Military Command, Guangzhou; ^2^Department of Orthopaedics, Xijing Hospital, Fourth Military Medical University, Xi'an; ^3^Department ofOncology, Guangzhou General Hospital of Guangzhou Military Command, GuangzhouChina

## Abstract

**Background and purpose:**

Experimental models of osteoporosis in rabbits are useful to investigate anabolic agents because rabbits have an active Haversian remodeling and achieve skeletal maturiaty quickly. In this study, an experimental model of osteoporosis in rabbits induced by a combination of ovariectomy and glucocorticoid was characterized to provide a useful model for prevention and therapy of osteoporosis.

**Methods:**

32 skeletally mature female rabbits were divided randomly into 4 groups: sham control, bilateral ovariectomy (OVX), 1mg/kg/day methylprednisolone (MP) for 8 weeks, and OVX in combination with MP. All rabbits were killed 10 weeks after surgery. Bone mineral density (BMD) of lumbar spine was measured by dual-energy X-ray absorptiometry at baseline, and at 6 and 10 weeks postoperatively. Bone microarchitecture and mechanical properties of lumbar vertebrae were investigated by high-resolution micro-computed tomography and compression test, respectively, after killing.

**Results:**

Mean BMD of the OVX+MP group at 10 weeks was reduced by about 36% from baseline (p < 0.001). Bone microarchitecture of lumbar vertebrae in the OVX+MP group indicated osteoporosis-associated deterioration. There was a statistically significant reduction in maximum load, stiffness, and energy absorption capacity of lumbar vertebrae in the OVX+MP group, but not in the OVX group, compared to the sham control. In the MP group, BMD and some microarchitecture parameters such as trabecular thickness and bone volume fraction were reduced. The mechanical properties were not statistically significantly different from those in the sham control group, however, although a negative trend was observed.

**Interpretation:**

Osteopenia can be induced experimentally in rabbits through a combination of OVX and MP, and can be evaluated by BMD, bone microstructure, and mechanical parameters.

## Introduction

As there is no ideal animal model for osteoporosis, since 1994 the US Food and Drug Administration (FDA) has required data from at least two animal species for preclinical evaluation of new experimental drugs (Thompson et al. 1995). Although the ovariectomized rat represents the “gold standard” in experimental animal models for postmenopausal osteoporosis studies (Kalu 1991, Frost and Jee 1992, Lelovas et al. 2008), it has several disadvantages, such as the failure to achieve true skeletal maturity, the lack a of Haversian system, and the low rate of remodeling in cortical bone (Rodgers et al. 1993, Mosekilde 1995). These conditions limit the use of rats for assessment of new treatments for osteoporosis, especially bone-forming agents, which require exploration of dynamic trabecular and cortical bone remodeling. Thus, it is necessary to characterize other experimental animal models for osteoporosis. Rabbits have often been used to study ingrowth of bone into implants and bone-implant interfaces (Mori et al. 1997, Southard et al. 2000, Cao et al. 2004). Rabbits achieve skeletal maturity at 7–8 months, show substantial intracortical remodeling, and have a more rapid bone turnover than other rodents and even primates (Gilsanz et al. 1988, Newman et al. 1995). In spite of these potential advantages, rabbit osteoporosis (OP) models have not been widely used and are not fully characterized (Newman et al. 1995, Mori et al. 1997). Castaneda et al. (2006, 2008) proposed an experimental model of OP in rabbits induced by a combination of ovariectomy (OVX) and glucocorticoid administration, but only bone mineral density (BMD) was determined. Without the assessment of microarchitecture and mechanics of the bone, a more complete skeletal characterization of the rabbit model cannot be achieved.

The aim of this study was to further characterize the experimental model of OP in rabbits induced by a combination of both experimental interventions (OVX and glucocorticoid administration). BMD, bone microarchitecture, and mechanical properties were determined for evaluation.

## Material and methods

### Animals

All animal care and experimental procedures were conducted with the approval of the Animal Care and Use Committee of Fourth Military Medical University, and were in accordance with the NIH Guidelines for the Care and Use of Laboratory Animals. 32 8-month-old (4.1 ± 0.38 kg body weight), skeletally mature, female New Zealand white rabbits were obtained from the Animal Center of Fourth Military Medical University. They were housed individually in stainless steel cages in a standard animal facility, where the room temperature was maintained at 21–24°C with 40–60% relative humidity and a 12-h light-dark cycle. The light cycle coincided with daylight hours. The standard chow (containing 0.8% calcium and 0.5% phosphorus) and tap water were available ad libitum. After a 10-day quarantine and acclimatization period, the baseline BMD of the lumbar vertebrae was measured in all animals. Based on the baseline data, the 32 rabbits were then randomly divided into 4 groups of 8 rabbits each (a sham control group, an OVX group, a methylprednisolone (MP) group, and an OVX+MP group).

### Experimental animal model of osteoporosis

**Figure 1.**
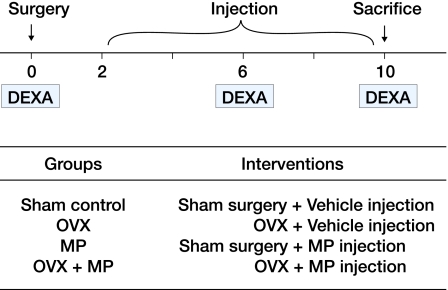
The experimental schedule and interventions in each group. OVX: ovariectomy; MP: methylprednisolone; DEXA: dual-energy X-ray absorptiometry.

A schedule of the experimental interventions is shown in Figure 1. Briefly, the rabbits were fasted for 24 h prior to surgery. The OVX group and the OVX+MP group received bilateral OVX through a ventral incision under general anesthesia with intramuscular injection of pentobarbital sodium (50 mg/kg). The sham control group and the MP group received sham surgery. All animals were fasted for another 12 h, and antibiotic prophylaxis with cefonicid sodium (100 mg/kg) was administered before and during the 5 days following surgery to minimize any complications. 2 weeks after the surgery, rabbits in the MP group and the OVX+MP group were injected intramuscularly with methylprednisolone succinate dissolved in 0.9% benzyl alcohol at a dosage of 1 mg/kg/day for 8 consecutive weeks; rabbits in the sham control and OVX groups were injected with 0.9% benzyl alcohol. Animals were weighed and the doses were adjusted on a weekly basis.

At the end of the above treatment, all animals were killed with a lethal dose of pentobarbital sodium. Lumbar vertebrae (L3 and L4) were dissected free, placed in sterile saline, and stored airtight at –20ºC until examination.

### Bone mineral density analysis

BMD of the lumbar spine was measured in vivo with a dual-energy X-ray absorptiometer (DEXA; Lunar DPX-IQ, Madison, WI) in all rabbits before the surgery; this was repeated 6 weeks postoperatively and immediately before killing (see Figure 1). Specific software for small animals was used. Under general anesthesia, each rabbit was fixed in the supine position on the stage of the DEXA, and a point located 3 cm below the navel was used as the external guide to focus the DEXA pencil beam at about L3–L4. Mean absorptiometry values of L3 and L4 were calculated.

### Micro-CT examination

A cone-beam-type desktop micro-CT system (Explore Locus SP, GE Healthcare) was used to quantify structural parameters of the lumbar vertebrae. The analytical conditions were 80 kV with 80 μA, and the spatial resolution was 14 μm according to the protocol of largetube_14 μm_150 min_SS for about 500 consecutive sections. For all vertebrae, a cylinder region of interest (ROI, diameter 3 mm × height 4 mm) was chosen and reconstructed three-dimensionally using Micview software. After thresholding, trabecular thickness (Tb.Th), trabecular number (Tb.N), trabecular separation (Tb.Sp), bone surface/bone volume (BS/BV), bone volume/total volume (BV/TV), and connectivity density (Conn.D) were determined. All evaluations were performed without any knowledge of the experimental identity of the sample.

### Mechanical testing

The specimens were thawed before mechanical tests and kept moist during all handling and test procedures. Both end-plates of the vertebral body were removed using a low-speed precision diamond saw with continuous irrigation to obtain parallel sections. The spinous, transverse, and articulate processes were also removed with the saw. By these procedures, a central part of the vertebral body with parallel ends was obtained, consisting of a central trabecular core and a compact shell. The individual vertebrae were then tested along the longitudinal axis in a materials testing machine (AGS-10kNG; Shimadzu Corporation, Japan) at a constant compression speed of 1 mm/min. The specimens were loaded to failure and maximum load, displacement, stiffness, and energy absorption capacity were recorded according to the load-displacement curves. Maximum load is the force (in N) that a material can sustain before failure. Displacement is the ultimate deformity before failure (in mm). The slope of the linear portion of the load-displacement curve (N/mm) was defined as stiffness. The area under the load-displacement curve was defined as energy absorption capacity (in mJ).

### Statistics

All statistical analyses were performed using commercially available software (SPSS version 10.0). Results are expressed as mean ± SD. Data from multiple groups were compared using one-way analysis of variance (ANOVA), and the significance of difference from the control group was determined by Dunnett post hoc test. Significant levels of all the tests were set at p < 0.05.

## Results

All animals survived the 10-week protocol and no surgical complications or macroscopic signs of infection were observed. The body weight of the 4 groups of animals ranged from 4.1 ± 0.25 to 4.3 ± 0.37 at baseline. Over the course of the study, body weight was similar in the experimental animals and the controls, and between groups (Table 1).

**Table 1.** Body weight (in kg) of the rabbits over the course of the study

Group	Baseline (before surgery)	Time after surgery		
		2 weeks	6 weeks	10 weeks
Sham control	4.09 (0.25)	4.13 (0.23)	4.11 (0.20)	4.11 (0.20)
OVX	4.29 (0.37)	4.25 (0.33)	4.35 (0.33)	4.38 (0.30)
MP	4.15 (0.23)	4.18 (0.21)	4.13 (0.20)	4.14 (0.23)
OVX+MP	4.20 (0.31)	4.19 (0.29)	4.25 (0.27)	4.28 (0.30)

Values are mean (SD). OVX: bilateral ovariectomy; MP: methylprednisolone.

### BMD measurements

Compared to the sham control, a significant reduction in BMD was seen in the OVX+MP group 6 weeks (p < 0.05) and 10 weeks (p < 0.001) after surgery, and BMD at the end of the experiment was reduced by 36% from baseline. On the other hand, the MP group did not show any remarkable changes of BMD until the endpoint, in comparison to the control group. BMD in the control group and in the OVX group remained stable throughout the study (Table 2).

**Table 2.** Variations in bone mineral density (BMD, mg/cm^2^) in rabbit lumbar spine with respect to baseline conditions and 6 and 10 weeks after intervention: OVX and/or methylprednisolone succinate (MP) administration

	Baseline	6 weeks after surgery		10 weeks after surgery	
Groups	BMD	BMD	ΔBMD [%]	BMD	ΔBMD [%]
Sham control	304 (39)	294 (30)	–10 (9) [–3.4]	307 (40)	3 (6) [1.0]
OVX	293 (31)	274 (23)	–19 (17) [–6.5]	268 (22)	–25 (24) [–8.5]
MP	296 (37)	267 (25)	–30 (23) [–9.9]	231 (33)	–65 (58) [–22] ^**a**^
OVX+MP	297 (36)	250 (23)	–46 (28) [–16] ^**a**^	189 (12)	–107 (26) [–36] ^**b**^

BMD values (mg/cm**^2^**) are mean (SD). ΔBMD means variations in BMD 6 or 10 weeks after intervention. Values in square brackets represent the percentage of change from baseline.

^**a**^ p <0.05) and

^**b**^ p <0.001 compared to sham control group.

### Bone microarchitecture

**Figure 2.**
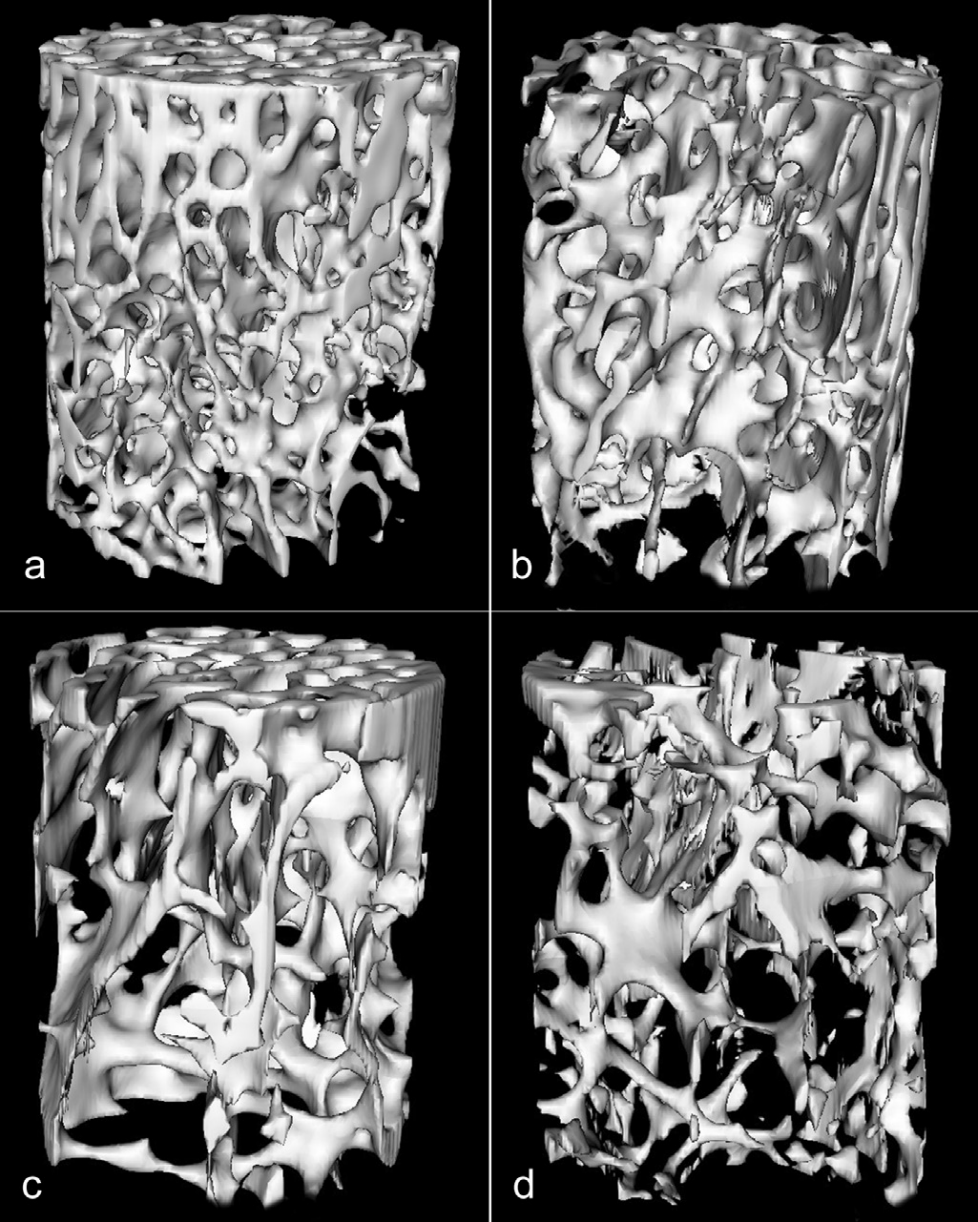
Three-dimensional reconstructions by micro-CT from lumbar vertebra of a sham control rabbit (panel a), a rabbit from the OVX group (panel b), a rabbit from the MP group (panel c), and a rabbit from the OVX+MP group. For data, see Table 3.

No differences in trabecular bone morphology were detected between the OVX group and control group (Table 3 and Figure 2). Rabbits in the MP group had less BV/TV (–17%, p < 0.05) compared to sham control group, with thinner trabeculae (–19%, p < 0.05). All structural parameters of the lumbar vertebrae in the OVX+MP group changed relative to the control group (Tb.N decreased by 24%, p < 0.05; Tb.Th decreased by 33%, p < 0.001; Tb.Sp increased by 58%, p < 0.01; BV/TV decreased by 39%, p < 0.001; BS/BV increased by 48%, p < 0.01; Conn.D. decreased by 35%, p < 0.01).

**Table 3.** Bone microarchitecture parameters of the lumbar vertebrae (micro-CT data) after the 10-week interventions. Values are mean (SD)

Groups	Trabecular number (1/mm)	Trabecular thickness (mm)	Trabecular separation (mm)	Bone volume/total volume (%)	Bone surface area/bone volume (1/mm)	Connectivity density (1/mm**^3^**)
Sham control	1.69 (0.24)	0.21 (0.03)	0.38 (0.07)	0.36 (0.03)	9.4 (1.6)	7.0(1.3)
OVX	1.74 (0.25)	0.19 (0.02)	0.39 (0.08)	0.36 (0.05)	10 (1.9)	6.6 (1.2)
MP	1.64 (0.37)	0.17 (0.02) ^**a**^	0.45 (0.13)	0.30 (0.04) ^**a**^	12 (2.6)	5.6 (1.5)
OVX+MP	1.28 (0.16) ^**a**^	0.14 (0.02) ^**c**^	0.60 (0.16) ^**b**^	0.22 (0.03) ^**c**^	14 (2.9) ^**b**^	4.5 (0.55) ^**b**^

^**a**^ p <0.05),

^**b**^ p <0.01), and

^**c**^ p <0.001 compared to the sham control.

### Mechanical properties of the lumbar vertebrae

In the OVX+MP group, maximum load was reduced by 39% (p < 0.001), stiffness was reduced by 40% (p < 0.01), and energy absorption capacity was 42% lower (p < 0.01) compared to the control group (Table 4).

**Table 4.** Mechanical parameters of lumbar vertebrae after the 10-week interventions. Values are mean (SD)

Group	Maximum load (N)	Displacement (mm)	Stiffness (N/mm)	Energy (mJ)
Sham control	1212 (153)	1.32 (0.36)	1091 (167)	850 (177)
OVX	1041 (111)	1.46 (0.39)	949 (190)	810 (91)
MP	984 (161) ^**a**^	1.28 (0.20)	838 (164)	696 (102)
OVX+MP	735 (87) ^**c**^	1.18 (0.24)	653 (204) ^**b**^	496 (73) ^**b**^

^**a**^ p <0.05),

^**b**^ p <0.01), and

^**c**^ p <0.001 compared to the sham control.

However, there was no statistically significant difference between the OVX and MP groups and the control group, except for the maximum load in the MP group (–19%, p < 0.05).

## Discussion

Rabbits have an active Haversian remodeling and achieve skeletal maturity quickly (Gilsanz et al. 1988). Ovariectomy and corticosteroid administration are the main treatments for establishment of experimental osteoporosis models in rabbits (Grardel et al. 1994, Southard et al. 2000, Eberhardt et al. 2001), but the induction of OP by any of these methods alone has been controversial. In our study, isolated OVX for 10 weeks did not induce adequate reduction of the BMD. This is in accordance with previously reported results (Mori et al. 1997, Turner et al. 2001), and suggests that the rabbit is a poor model for ovarian deprivation osteoporosis. Since a few reports have indicated that systemic corticosteroids at high dose may result in cartilage damage and even death (Warman and Boskey 1983, Grardel et al. 1994), we chose a dose of 1mg/kg/day that has been found to have an effect on bone turnover without any additional detrimental effects (Eberhardt et al. 2001, Castaneda et al. 2008). At the same time, we tested the combination of ovariectomy and corticosteroid treatment to assess whether a more consistent model of osteoporosis could be established in this way. To our knowledge, no authors have reported an adequate reduction in BMD in rabbits according to the WHO criteria for osteoporosis in humans (Lill et al. 2002). Human osteoporosis is defined as a reduction in BMD of more than 2.5 SD, which is approximately 25%. In this study, OVX+MP induced a decrease in BMD of about 36%.

Rabbits become sexually mature between 20 and 24 weeks of age and reach skeletal maturity at 28–32 weeks (Wang et al. 2006). The peak bone mass occurs at 32–36 weeks (Gilsanz et al. 1988, Norris et al. 2001) and regular bone resorption and formation is well established at this stage. In our study, the bone mass in sham control rabbits remained stable, indicating that in contrast to other rodents, rabbits can reach true skeletal maturity.

High-resolution micro-computed tomography (micro-CT) can be used to measure both the quantity and quality of trabecular bone (Mittra et al. 2008). In our study, the lower BMD found in the lumbar vertebrae of the MP group and the OVX+MP group was reflected by structural changes in the high-resolution micro-CT image. The micro-CT analysis showed that the bone volume fraction (BV/TV) and the trabecular thickness in the MP-group rabbits decreased as compared to the sham control animals. All structural parameters (Tb.N, Tb.Th, Tb.Sp, BV/TV, BS/BV, Conn.D) of the lumbar vertebrae in OVX+MP-group rabbits had statistically significant differences compared to the control group, suggesting that microarchitectural deterioration of the lumbar vertebrae had occurred in the rabbits.

Compression testing of lumbar vertebrae is recommended for testing the mechanical properties of cancellous bone (Adinoff and Hollister 1983). Maximum load is a widely used parameter for mechanical evaluation, and represents the maximum force that a material can sustain before failure. Displacement, stiffness, and energy absorption capacity are also important (Peng et al. 1994, Gurgul et al. 2008). Bone fragility can be defined by mechanical parameters, including maximum load, displacement, and energy absorption (Turner 2002). In the present study, we found that maximum load and stiffness were reduced in the OVX+MP group (Table 4), indicating that induction by OVX and MP for 10 weeks could reduce bone strength. Displacement was reduced by 10% in the OVX+MP group, although no statistically significant difference was found. The energy absorption capacity (work to fracture) of the OVX+MP group was reduced relative to the control group. These results suggest that bone fragility was increased and that the bone was more susceptible to fracture in the OVX+MP group.

In the rabbit study of Castaneda et al. (2008), the BMD of the lumbar vertebrae decreased by 17% from baseline at 6 weeks. In our study, a longer induction time was used and we found a decrease in BMD of about 36%. Our modified rabbit model is characterized by low bone mass and microarchitectural deterioration in bone tissue, with impaired skeletal strength and increased susceptibility to fracture.

The search for an experimental model of osteoporosis will continue as long as there are discrepancies between the results in humans and in potential models. Our model of osteoporosis in rabbits, induced by a combination of OVX and glucocorticoid administration, has several advantages over other animal models, as it is a model in a skeletally mature mammal that is easy to obtain and care for, and the induction procedure is relatively short and uncomplicated, with a high degree of reproducibility. In addition, we believe that it would be more appropriate to involve BMD, bone microarchitecture, and mechanical parameters in evaluation of models of osteoporosis.
